# New Insight into the Role of Adenosine in Demyelination, Stroke and Neuropathic Pain

**DOI:** 10.3389/fphar.2020.625662

**Published:** 2021-01-29

**Authors:** Elisabetta Coppi, Ilaria Dettori, Federica Cherchi, Irene Bulli, Martina Venturini, Felicita Pedata, Anna Maria Pugliese

**Affiliations:** Department of Neuroscience, Psychology, Drug Research and Child Health (NEUROFARBA), Section of Pharmacology and Toxicology, University of Florence, Florence, Italy

**Keywords:** cerebral ischemia, oxygen-glucose deprivation, adenosine receptors, oligodendrocyte differentiation, neuropathic pain, dorsal root ganglion neurons, neuroinflammation

## Introduction

Our group of research has a long-time experience in studying the role/s of adenosine P_1_ receptors in health and disease conditions of the central nervous system (CNS). The effects of adenosine were investigated in our laboratory by the use of *in vitro* or *in vivo* models.

In most recent years, main topics of our research concerned the study of adenosine and its receptors in the physiological processes of myelination and in pathological conditions of ischemia. Most recently, the group addressed the study of adenosine-mediated mechanisms in pain control.

Here, we are going to summarize our recent findings.

## Areas of Interest

### Role of A_2A_Rs and A_2B_Rs in Oligodendrogliogenesis

Oligodendrocytes (OLs) are the only myelinating cells in the CNS and differentiate throughout adult life from their progenitors, the oligodendrocyte progenitor cells (OPCs), to produce myelin sheets around neuronal axons. Growing evidence indicates that failure of myelin formation in demyelinating diseases, i.e., multiple sclerosis (MS), arises from the disruption of OPC differentiation process (Levine et al., 2001). Hence, therapeutic strategies aimed at fostering this process are largely attractive.

Purines are important modulators of cell development in different cellular systems (Coppi et al., 2007b; Coppi et al., 2012; Burnstock and Dale, 2015). Concerning oligodendrogliogenesis, it is now generally recognized that adenosine plays a key role in OPC maturation. All subtypes of adenosine P1 receptors are expressed during the entire process of OPC maturation (Stevens et al., 2002; Coppi et al., 2013a; Coppi et al., 2020a). Indeed, adenosine is known to be released upon electrical stimulation of nearby neurons to acts as a neuron-glial transmitter that inhibits OPC proliferation and facilitates their differentiation (Stevens et al., 2002). Adenosine A_1_R stimulation is also known to promote OPC migration (Othman et al., 2003), another important process aimed to repair brain damage and repopulate the injured site (de Castro and Bribian, 2005).

Our attention was devoted to study the role of the different subtypes of adenosine P_1_ receptors, the adenosine A_2A_Rs and A_2B_Rs, in this process. Our group of research significantly contributed to gain insight into the field by demonstrating that selective adenosine A_2A_R stimulation inhibits OPC maturation by reducing voltage-dependent, sustained, outward K^+^ currents (I_K_) sensitive to tetraethyilammonium (TEA) (Coppi et al., 2013a; Coppi et al., 2015; Cherchi et al., 2020). These data are well in keeping with original research from Gallo and coworkers who first demonstrated that TEA, incubated in OPC cultures, reduces the expression of myelin proteins (Gallo et al., 1996).

Successive studies by our laboratory demonstrated that adenosine A_2B_R agonists, either the prototypical compound BAY60-6583 or the recently synthetized BAY60-6583-analogue P456 (Betti et al., 2018), not only inhibit sustained I_K_ but also transient I_A_ conductances in cultured OPCs (Coppi et al., 2020a). The effect on I_K_ was mimicked and occluded by forskolin, demonstrating its dependency upon intracellular cAMP increase, consistently with Gs-coupling of A_2A_R and A_2B_R and with the fact that the Gi-coupled GPR17 (Pugliese et al., 2009b) elicits the opposite effect in OPCs (Coppi et al., 2013b). Importantly, in the same work, we reported that A_2B_R agonists activate sphingosine-kinase-1 (SphK1), an ubiquitous enzyme responsible for the formation of sphingosine-1-phosphate (S1P). Finally, *in vitro* silencing of A_2B_R was sufficient to increase OPC maturation, to inhibit SphK1 expression, and to strikingly rise the levels of S1P lyase, the enzyme devoted to irreversible degradation of S1P. In conclusion, our data demonstrate that A_2B_R stimulation enhances intracellular S1P levels, whereas A_2B_R silencing triggers S1P catabolism. Since S1P acts as a mitogen in a variety of cells including OPCs (Jung et al., 2007), we postulate that the antidifferentiative role of A_2B_R in oligodendrogliogenesis is exerted by increasing intracellular S1P levels. These results are particularly attractive since the first approved oral agent for MS treatment is the S1P analogue fingolimod (FTY720). Thus, our results may open interesting possibilities about a pharmacological control of myelin formation and repair during demyelinating pathologies by A_2B_R ligands, possibly in combination with S1P modulators.

### Role of Adenosine A_2A_Rs and A_2B_Rs in Brain Ischemia

A main topic of our research group concerns the role of different P1 receptor subtypes in modulating the damage inflicted to neurons and glia by ischemia. To this purpose, both the *in vitro* model of oxygen and glucose deprivation (OGD) in rat hippocampal slices or the *in vivo* model of ischemia induced by middle cerebral artery occlusion (MCAo) in the rat were used. It is well accepted that adenosine released during brain ischemia exerts a neuroprotective role by A_1_R-mediated mechanisms (Pedata et al., 2016). However, as A_1_R-selective agonists became clinically less and less attractive due to important side effects such as bradycardia and sedation (Sollevi, 1986; Vonlubitz et al., 1994), our research focused on A_2A_R and A_2B_R subtypes.

In the *in vitro* OGD model in acute rat hippocampal slices, we monitored the excitotoxic damage mediated by excessive glutamate release during a severe OGD insult of 7 or 9 min (Pugliese et al., 2006; Pugliese et al., 2009a; Colotta et al., 2012). Purines are important modulators of neuronal damage during OGD (Coppi et al., 2007a; Maraula et al., 2014). Selective antagonists of adenosine A_2A_Rs, the SCH58261 and SCH442416, protect from the OGD-induced irreversible disappearance of field excitatory post-synaptic potentials (fEPSPs) and from the appearance of anoxic depolarization (AD), an unequivocal sign of neuronal injury. The A_2A_R antagonists also delay AD onset invariably induced during a long period, 30 min, OGD in the CA1 region (Pugliese et al., 2009a) or in the dentate gyrus (DG) (Maraula et al., 2013) of the hippocampus. In the DG, A_2A_R antagonists also restore the number of 5-bromo-20-deoxyuridine-positive (BrdU^+^) newborn neurons 6 h after the end of the insult (Maraula et al., 2013), limit the extent of CA1 damage to neurons and glia assessed by propidium iodide permeability, and reduce astrocyte activation (Pugliese et al., 2009a).

Similar results were later found with antagonists to the “partner” adenosinergic receptor, i.e., the A_2B_R subtype. The compounds MRS1754 and PSB603, by blocking the A_2B_R, protected CA1 hippocampal slices from OGD-induced damage by preventing irreversible synaptic failure and AD appearance induced by OGD and counteracted CA1 neuronal loss, astrocyte activation, and cytochrome C release (Fusco et al., 2018). In line with previous literature (Goncalves et al., 2015), we confirmed that protection by the adenosine A_2B_R antagonists is attributable to diminished glutamate release since the A_2B_R agonist BAY60-6583, and its analogue P456, significantly decreased hippocampal paired pulse facilitation (PPF) (Fusco et al., 2019), an electrophysiological paradigm used to detect presynaptic neuromodulation of glutamate release (Regehr, 2012).

Consistent with previous results, we found that the selective A_2A_R antagonist, SCH58261, acutely (5 min) or subchronically (5 min, 6 h, and 20 h) administered in the *in vivo* model of permanent MCAo (pMCAo) in the rat, 24 h thereafter, was protective against neurological deficit and brain damage (Melani et al., 2003; Melani et al., 2006; Melani et al., 2009). Such protection can be related to the activation of deleterious pathways of MAPK expressed in microglia, such as p38, or JNK, expressed in mature OLs and in OPCs (Melani et al., 2006), which is considered a inhibitory molecule that can hinder myelin reconstitution and neuron functionality (Melani et al., 2009). Such a protective effect induced by adenosine A_2A_R antagonism in the first hours after ischemia found a valuable explanation in the ability to control excessive glutamate extracellular concentrations as detected by the technique of cerebral microdialysis (Melani et al., 2003) and the ensuing acute excitoxicity after ischemia.

In an apparent paradoxical manner, we found that, in the model of transient, 1 h, MCAo (tMCAo), not the antagonist but the agonist at the A_2A_R, CGS21680, exerted protective effects. However, this observation was made later from ischemia induction, i.e., 7 days after ischemia. Indeed, the chronic administration (twice/day for 7 days) with CGS21680 induced protection from neurological deficit, weight loss, cortical infarct volume, myelin disorganization, and glial activation (Melani et al., 2014). Without excluding that protection by A_2A_R agonists is attributable to central effects, such as increasing expression and release of neurotrophic factors (Sebastiao and Ribeiro, 2009), data reported by our research group point toward a protective effect due to peripheral A_2A_R activation. A_2A_Rs are expressed on blood cells where they definitely reduce adhesion cell factor production, platelet aggregation and neutrophil activation, exerting, therefore, an antithrombotic, antioxidant and anti-inflammatory effect. Accordingly, two days after tMCAo, chronic treatment with CGS21680 reduced the number of infiltrated blood cells in the ischemic areas (Melani et al., 2014).

Most recently, we found similar results by studying the effect of BAY60-6583 (administered twice/day for 7 days in the tMCAo model), a selective agonist of the other A_2_R subtype, that the A_2B_R in most cases is coexpressed with A_2A_Rs on hematic cells where it inhibits vascular adhesion (Yang et al., 2006) and migration of inflammatory cells (Wakai et al., 2001; Konrad et al., 2012). Two days after ischemia, the A_2B_R agonist reduced blood cell infiltration in the ischemic cortex (Dettori et al., 2020). Interestingly, 7 days after ischemia, the A_2B_R agonist also decreased TNF-α and increased IL-10 levels in the blood (Dettori et al., 2020). Both factors are considered valuable blood markers of the brain damage following an ischemic insult (Jickling and Sharp, 2011). These results stress the key research questions of the predictive value of blood biomarkers in stroke.

By and large, results underlie that, after hypoxia/ischemia, brain injury results from a complex sequence of pathophysiological events that evolve over time: a primary acute mechanism of excitotoxicity and periinfarct depolarizations followed by a secondary brain injury activation triggered by protracted neuroinflammation (Coppi et al., 2020b). Information acquired up to now indicate that adenosine A_2_Rs located on any cell type of the brain and on vascular and blood cells partake in either salvage or demise of the tissue after a stroke. Thus, they all represent important targets for drugs having different therapeutic time-windows after stroke.

### Role of Adenosine A_3_Rs in Pain Control

Most recently, our group addressed the study of adenosine-mediated mechanisms of pain control. It is known that agonists at A_3_R subtype are effective pain suppressors in animal models of chronic constriction injury, chemotherapic pain (Janes et al., 2016), and also in colitis-induced visceral hypersensitivity (Lucarini et al., 2020). Their safe pharmacological profile, as shown by clinical trials for other pathologies, i.e., rheumatoid arthritis, psoriasis, and cancer, confers a realistic translational potential, thus encouraging research studies on the molecular mechanisms underpinning their antinociceptive actions. A number of pathways, involving central or peripheral mechanisms, have been proposed.

Our group recently demonstrated that the prototypical A_3_R agonist Cl-IB-MECA and the new, highly selective, A_3_R agonist MRS5980 (Tosh et al., 2014) inhibit the neuronal N-type voltage-dependent Ca^2+^ current in DRG neurons, a known pain-related current, more efficiently than the A_1_R agonist CPA (Coppi et al., 2019). Indeed, current-clamp experiments confirmed that Cl-IB-MECA significantly decreased the DRG neuronal firing (Coppi et al., 2019).

Our findings contributed to unveil one of the mechanisms of A_3_R-based pain control and reinforce the concept of A_3_R agonists as novel, promising, nonnarcotic agents for chronic pain relief.

## Discussion

On the whole, our recent findings support the notion of adenosine receptors being involved in a number of central and peripheral nervous system diseases, from stroke and demyelination to neuropathic pain. Beyond the well-known protective effect of A_1_Rs, whose agonists are unfortunately not devoid of side effects, A_2_R- and A_3_R-selective ligands are attractive tools to develop innovative therapeutic strategies. Indeed, A_2_R antagonists exert significant neuroprotection in the initial (within 24 h) postischemic damage in the brain due to inhibition of glutamate excitotoxicity. Additional neuroprotection by A_2A_R and/or A_2B_R antagonists could be due to a promyelinating effect exerted by these compounds by stimulating OPC differentiation. On the other hand, at later phases (i.e., 7 days) after stroke, A_2_R agonists may attenuate neuroinflammation and immune cell infiltration to reduce brain damage. Finally, recent results emerging from our group revealed one of the mechanisms responsible for A_3_R-mediated pain control, i.e., inhibition of N-type Ca^2+^ currents and electrical activity in primary sensory neurons of the DRG, thus supporting these receptors as innovative nonopioid compounds for the treatment of chronic pain.

## Author Contributions

EC, ID, AMP, and FP wrote the paper. FC, EC, ID, MV, and IB performed and analyzed experiments described in the paper. AMP, EC, and FP provided funding. AMP and FP supervised the work.

## Funding

The study was supported by the University of Florence (Fondi Ateneo Ricerca) to AMP, the Ministry of Education, Universities and Research, Italy: MIUR-PRIN 2017CY3J3W_003 to FP, Fondazione Umberto Veronesi to EC (FUV 2020-3299), and Fondazione Italiana Sclerosi Multipla (FISM): 2019/R-Single/036 (AMP) to AMP and EC.

## Conflict of Interest

The authors declare that the research was conducted in the absence of any commercial or financial relationships that could be construed as a potential conflict of interest.
